# Investigation of Staphylococcus lugdunensis and Selected Coagulase Negative Staphylococci Isolated from Blood Culture bottles and Determination of their Sensitivities to Antibiotics

**DOI:** 10.12669/pjms.38.3.4738

**Published:** 2022

**Authors:** Aynur Veliev, Yasar Nakipoglu

**Affiliations:** 1 Aynur Veliev, Istanbul Faculty of Medicine, Department of Medical Microbiology, Istanbul University, Istanbul, Turkey; 2 Yasar Nakipoglu, Istanbul Faculty of Medicine, Department of Medical Microbiology, Istanbul University, Istanbul, Turkey

**Keywords:** PYR, ODC, *Staphylococcus lugdunensis*, Blood culture, Coagulase-negative staphylococci, Antibiotic susceptibility test

## Abstract

**Objectives:**

Coagulase-negative staphylococci (CNS) are commensal skin microbiota but may also cause septicemia, endocarditis, and systemic infections. *Staphylococcus lugdunensis*, is a member of CNS, but their antibiotic susceptibility test should be evaluated as *Staphylococcus aureus* not as CNS. We aimed to investigate *S.lugdunensis* and selected CNS strains by simple biochemical method and determination of their susceptibilities to antibiotics.

**Methods:**

A total of 251 CNS isolates were collected from blood culture bottles sent to Istanbul Faculty of Medicine Department of Medical Microbiology, between 2018 and 2019. PYR (pyrrolidonyl arylamidase) and ODC (ornithine decarboxylase) tests were performed on total of CNS isolates and API Staph was used for identification of the isolates giving positive result in both or either of these two tests. Disk diffusion method was used for the determination of antibiotic susceptibility of the isolates. *S. aureus* ATCC 25923 and S *S.lugdunensis* ATCC® 49576 strains were used as quality control strains in disc diffusion method, and biochemical tests, respectively.

**Results:**

Twenty three out of 251 CNS isolates were positive in each or both of PYR and ODC tests. We detected the first *S.lugdunensis* isolate from eye vitreous fluid of patient developed a postoperative endophthalmitis in Turkey. This isolate gave dual positive with ODC, PYR, and API Staph. Other 22 CNS isolates were from blood cultures and distributed as follows; 14 *Staphylococcus haemolyticus* and three *Staphylococcus chromogenes* isolates were PYR positive and ODC negative and five *Staphylococcus epidermidis* isolates were ODC positive and PYR negative. All isolates except *S.lugdunensis* were resistant to penicillin (95.7%) and 20 (87.0%) isolates were found to be methicillin resistant.

**Conclusions:**

ODC and PYR are cost effective tests and easily applicable for accurate identification of *S.lugdunensis*, and eliminating of opportinistic pathogens such as *S. epidermidis*, *S. haemolyticus*, and *S. chromogenes* from other CNS species in postoperative endophthalmitis and pateints with malignancies. Linezolid was very effective (100%) on four selected CNS species.

## INTRODUCTION

Coagulase-negative staphylococci (CNS) are often considered commensal bacteria and they are members of the skin microbiota in humans.^1^As far, there are 48 species of CNS and some of them cause nosocomial bloodstream infections.^1^ *Staphylococcus lugdunensis* is a very particular CNS and closer to *Staphylococcus aureus* than other CNS in terms of virulence and emerged as an important pathogen causing endocarditis and skin/soft tissue infections.^2^,^3^ Clinical laboratory standards institute (CLSI) and the European Committee on Antimicrobial Susceptibility Testing (EUCAST) reported that the evaluation of antibiotic susceptibility test (AST) of *S.lugdunensis*should be done as *S.aureus*, not as CNS.^4^,^5^ The virulence factors of *S.lugdunensis* are also very closer to *S.aureus* than other CNS.^3^-^5^ Therefore, it is important to identify *S.lugdunensis* and differentiate it from other CNS species to avoid incorrect AST result and treatment failure. Although there are various automated identification systems such as Microscan Pos Combo Panel, Phoenix (PHX), and Vitek 2 for the identification of CNS species, these systems are only reliable in big diagnostic laboratories. *S.lugdunensis* is the only species that gives a positive result with dual PYR (pyrrolidonyl arylamidase) and ODC (ornithine decarboxylase) tests among CNS species.^3^Yen Liu et al.^6^, in their review published in 2010, mentioned that *S.lugdunensis* had a serious infective endocarditis effect and they advised to use PYR and ODC tests for identification of this bacterium.A study reported by Celebi et al.^7^ referred that early cardiac surgery should always be considered in the treatment of infective endocarditis due to *S.lugdunensis*. *Staphylococcus epidermidis* gives a variable result with ODC and the rest are negative.^3^ *S.epidermidis* is by far the most prevalent CNS in microbiological samples and the primary cause of CNS-related infections, particularly in nosocomial setting, endophthalmitis vitrectomy and in patients with artificial heart valves or with intravenous catheters commonly used in hospitals.^8^ *Staphylococcus haemolyticus* is a commensal bacterium but is also a frequent nosocomial pathogen that has been described mainly in catheter-related bacteremia, blood and also the second most frequently isolated CNS from clinical cases after *S. epidermidis*.^8^ *Staphylococcus chromogenesis* colonizing HIV-positive patients and causing bloodstream infections in patients with AIDS.^9^,^10^

According to CLSI, the antibiotic susceptibility test (AST) of *S.lugdunensis* should be performed and evaluated as *Staphylococcus aureus*, not as CNS, therefore, identification of *S.lugdunensis* is important especially for isolates of serious clinical samples such as blood, catheters, eye vitreous fluid to avoid incorrect AST and treatment failure. In our study, we aimed to investigate *S. lugdunensis*, and other CNS strains such as *S.epidermidis, S.haemolyticus*,and *S.chromogenes* and determine their susceptibilities to different antibiotics.^11^

## METHODS

### Bacterial isolates

Between 2018-2019, 7400 blood culture bottles were examined at the Department of Medical Microbiology, Istanbul Medical Faculty Istanbul, Turkey. Two hundred fifty one cultures (3.4%) were revealed growth of colonies chracterized as Gram-positive cocci, catalase-positive,and tube Plasma Coagulase (BD BBL, Diamed, Turkey) negative and defined as CNS.*S.lugdunensis* ATCC 49576 strain was used as a positive control in ODC and PYR tests and *S. aureus* ATCC 25923 strain was used as control strain in disc diffusion method. All strains were stored at -80 °C.

All stored CNS strains were cultured on tryptic soy agar (TSA, BD, France) at 36 ± 2 °C after an average of 18-24 hours incubation time. For this study, the Istanbul University Faculty of Medicine Ethics Committee approval (No: 2019/88) was obtained.

### ODC Test

In this study, the pH of the MIO medium which is used to identify Gram-negative bacteria was modified and adjusted from 6.5± 0.2 to 5.6 ± 0.2 for *S. lugdunensis*.CNS isolates were inoculated in MIO media and evaluated after 24 hours of incubation at 36 ± 2 ° C. The purple color was evaluated as positive and yellow color as a negative test result. *S. lugdunensis* ATCC 49576was used as a positive control strain and the previously identified *S.epidermidis* isolate as a negative control.^3^

### PYR Test

PYR test (ChemBio, Turkey) was carried out following the manufacturer’s recommendations. *S. lugdunensis* ATCC49576 was used as a positive control strain, and *S.epidermidis* was used as a negative control.^3^

### Identification with API Staph

In case of observing positivity in any of ODC and PYR tests API Staph (bioMerieux, France)was performed for identifying the species of CNS. The test was performed according to the manufacturer’s instructions.

### Antibiotic Sensitivity Tests (AST)

Kirby-Bauer disk diffusion method was applied and its interpretation was made according to CLSI standards. The following disks were used: Clindamycin (CC, 2 µg), erythromycin (E, 15 µg),cefoxitin (FOX, 30 µg), gentamicin (GN, 10 µg), levofloxacin (LEV, 5 µg), linezolid (LZD, 30 µg), penicillin (P, 10 µg), trimethoprim-sulfamethoxazole (SXT, trimethoprim 1.25 µg, sulfamethoxazole 23.75 µg) and telithromycin (TEL, 15 µg).*S.aureus* ATCC® 25923 was used as quality control strain in the disc diffusion method. Methicillin resistance in *S.lugdunensis* was evaluated as *S.aureus* according to CLSI whereas other CNS strains were evaluated as CNS.^11^

## RESULTS

### We performed ODC and PYR tests for all 251 CNS strains which isolated from blood culture bottles and the results are given below:

### ODC and PYR:

*ODC and PYR positive CNS isolates:* One isolate (0.4%) from the vitreous fluid of a patient with postoperative endophthalmitis was identified as *S.lugdunenesis* and confirmed with API Staph in parallel with *S.lugdunensis* ATCC® 49576 control strain.

*ODC positive and PYR negative isolates:* Five (2%) were found to be ODC positive and PYR negative. These isolates were identified as *S.epidermidis* with API Staph.

*PYR positive and ODC negative isolates:*Seventeen isolates (6.8%) were found to be PYR positive and ODC negative. In the identification of these isolates with API Staph, 14 were *S.haemolyticus* (5.6%) and three were *S.chromogenes* (1.2%).

*PYR and ODC negative isolates:* 228 (90.8%) isolates were identified as PYR and ODC negative. These isolates were excluded from the scope of the study for *S.lugdunensis* and other selected species of CNS. Therefore, determination of species and antibiotic susceptibility tests were not performed on these isolates.

### Disk diffusion method:

Disc diffusion method was applied on 23 clinical isolates, in which 14 were *S.haemolyticus*, five were *S. epidermidis*, three were *S.chromogenes*, and one was *S.lugdunensis*. Only three isolates were susceptible to methicillin (strain no 31 *S.epidermidis*, strain no 45 *S.lugdunensis*, strain no 237 *S.chromogenes*,).The most effective antibiotics in our study were as follows; Linezolid (100%), telithromycin(69.6%), gentamicin (34.8%), each of levofloxacin and clindamycin (21.7%), trimethoprim-sulfamethoxazole (13.0%), erythromycin (8.7%), and penicillin (4.3%) (Table-I).

**Table I:** Antibiotic susceptibility test results of 23 species of coagulase-negative staphylococcal isolated from blood culture bottles.

Isolate no	Bacterial species	FOX	P	E	CC	LEV	SXT	GN	TEL	LZD
2	*S. haemolyticus*	R	R	R	R	R	R	R	I	S
4	*S. haemolyticus*	R	R	R	R	R	R	R	S	S
8	*S. haemolyticus*	R	R	I	R	R	I	R	S	S
11	*S. chromogenes*	R	R	R	R	R	R	I	R	S
17	*S. haemolyticus*	R	R	I	R	R	I	R	S	S
20	*S. haemolyticus*	R	R	R	R	R	I	R	I	S
30	*S. epidermidis*	R	R	S	S	R	R	S	S	S
31	*S. epidermidis*	S	R	R	S	S	S	S	S	S
32	*S. epidermidis*	R	R	R	I	S	S	S	S	S
45	*S.lugdunensis*	S	R	S	S	S	S	S	S	S
49	*S. haemolyticus*	R	R	R	R	R	I	R	S	S
55	*S. haemolyticus*	R	R	R	R	I	I	R	R	S
86	*S. epidermidis*	R	R	R	R	R	I	S	R	S
94	*S. haemolyticus*	R	R	R	R	R	R	R	R	S
115	*S. epidermidis*	R	R	R	R	R	I	R	S	S
124	*S. haemolyticus*	R	R	R	R	R	I	S	R	S
148	*S. haemolyticus*	R	R	R	R	S	R	I	S	S
160	*S. haemolyticus*	R	R	R	R	R	I	R	S	S
174	*S. chromogenes*	R	R	R	S	R	R	S	S	S
181	*S. haemolyticus*	R	R	R	I	R	R	R	S	S
202	*S. haemolyticus*	R	R	R	R	R	I	R	S	S
218	*S. haemolyticus*	R	R	R	R	R	I	R	S	S
237	*S. chromogenes*	S	R	R	S	S	R	S	S	S

**Abbreviations:**CNS: CC: Clindamycin, E:Erythromycin, FOX: Cefoxitin, GN: Gentamycin, LEV: Levofloxacin, LZD: Linezolid, P: Penicillin, SXT: Trimethoprim-Sulfamethoxazole, TEL: Telithromycin, R; Resistance, I: Intermediately susceptible, S: Susceptible.

### The characteristics of patients infected with selected species of CNS

The clinical characteristics of patients infected with 23 isolates to total 251 patients are shown in Table-II. One *S.lugdunensis* strain from eye vitreous fluid and other 22 strains (14 *S.haemolyticus* five *S.epidermidis* and three *S. chromogenes)*were isolated from blood cultures. All 23 strains were sensitive to linezolid and 20 were resistant to methicillin.

**Table II:** The clinical features of 23 patients infected with S.lugdunensis, Staphylococcus haemolyticus, Staphylococcus epidermidis, and Staphylococcus chromogenes isolates.

Coagulase negative staphylococci (n: 23)	Clinical feature of patients infected with coagulase negative staphylococci (CNS)(number ofgrowth positive patient/ total number of CNS)
S.lugdunensis(n:1)	Vitreous fluid of a patient with postoperative endophthalmitis (1/1)
S. haemolyticus(n:14)	Febrile(1/22)
	Metabolic disorder (1/9 )
	Epilepsy (1/7)
	Chronic Lymphocytic Leukemia (CLL) (1/5)
	Kidney diseases (1/4)
	Chronic Myeloid Leukemia (CML) (1/3)
	Phenylketonuria (1/2)
	Hormonal disorder (1/1)
	Diabetic insipidus (1/1)
	Hodgkin lymphoma (1/1)
	Nephrotic syndrome (1/1)
	Acute Lymphoblastic Leukemia (ALL) (1/1)
	Cerebral palsy (1/1)
	Traffic accident (1/1)
S. epidermidis (n:5)	Febrile (1/22)
	HCV cirrhosis (1/2)
	Langerhans cell histiocytosis (1/1)
	Acute Myeloid Leukemia (AML) (1/1)
	Parkinson’s disease (1/1)
S. chromogenes(n:3)	Febrile (1/22)
	Malignancies (1/7)
	lung cancer (1/4)

### One S.lugdunensis

Strain no 45 was isolated from vitreous fluid of a postoperative endophthalmitis of 75 year old woman . The strain was susceptible to methicllin and other tested antibiotics but resistant to penicillin.

### Three S. chromogenes isolates

All three strains (11, 174, 237) were resistant to methicillin but strain no 11 was most resistant than other two strains. This strain isolated from 65-year-old who had lung cancer, and other two strains isolated from patient with hematological malignancy and febrile patient, respectively.

### Five S.epidermidis isolates

Strain no 31 infected 54 year Parkinson patient and was susceptible to methicillin and erythromycin, and other four (30, 32, 86, 115) strains were resistant to methicillin and most antibiotics and isolated from 56 years old with acute myeloid leukemia (AML), 72 years old patient with hepatitis C cirrhosis, three years old patient with langerhans cell histiocytosis, and five month old febrile patient.

### 14 S.haemolyticus isolates

All strains were resistant to methicillin. Linezolid , telithromycin, and trimethoprim-sulfamethoxazole were the most effective antibiotics. These strains infected patients old ranged between three months-73 years with different clinical characteristics (Table I and II).

## DISCUSSION

CLSI and EUCAST emphasized the necessity of AST of *S.lugdunensis* because this species unlike other CNS being resembles *S.aureus*,not CNS (4,5).^4^,^5^ *In the present study, 251 CNS strains were isolated from blood culture bottles*. PYR and ODC tests successfully identified *one (0.4%) S.lugdunensis* and differentiated 22 *isolates from other CNS which included*,14 (5.6%) *S.haemolyticus*, five (2.0%) *S.epidermidis,and* three (1.2%) *chromogenes*. *S.lugdunensis* (isolate no 45) was isolated from the vitreous fluid of a patient with postoperative endophthalmitis and this is the first isolate from Turkey. Accurate AST was done according to CLSI. *I*t should not be forgotten that rapid and appropriate antibiotic use is very important in the infections with *S*. *S.lugdunensis* because it has an ability to form biofilm in six hours which prevents reach of the antibiotics to their target.^12^ *From this point of view, many life-threatening S.lugdunensis infections could be treated with penicillins and cephalosporins when correctly identified. S.lugdunensis in our study was found to be susceptible to methicillin and all tested antibiotics except penicillin. Five* studies have been reported from Turkey on *S.lugdunensis* and selected CNS species by using API Staph, automated Vitek 2, Phoenix system, and 16 S RNA Polymerase Chain reaction (PCR). Koksal et al.^13^ reported a study on 200 CNS blood isolates. Their rate of CNS species except for *S.chromogenes* (1.5%) was higher compared with our rates. Among these species, *S.epidermidis* (43.5%), *S.haemolyticus*(11.5%)*, S.lugdunensis* (9.0%), and methicillin resistance was detected in 67.5% isolates.^13^ We detected that *S.haemolyticus* (5.6%), was the predominant species followed by *S.epidermidis* (2%), *S.chromogenes* (1.2%), and all our 20 blood isolates were found to be resistant to methicillin . Yazgi et al.^14^ isolated methicillin susceptible *S.lugdunensis* strain from wound culture. They identified the bacterium by ODC and PYR tests and confirmed it by API Staph, and other automated systems. In opposite to this strain, our strain was resistant to penicillin. Kivanc et al.^15^ isolated 12 *S.lugdunensis* ( 14.4% ) out of 83 CNS strains from surface of eye conjounctival of diabetic patients identified by Vitek 2 system. Nine were resistant to methicillin and two of them were found to be strong biofilm producers. Celebi et al.^7^ reported the first case of penicillin resistant *S.lugdunensis* endocarditis from Turkey in 2009. Dundar et al.^16^ reported 41 *S.lugdunensis* isolates most of them were skin soft tissue .They identified the isolates by conventional method and confirmed them by 16S rRNA gene sequencing. All were methicillin susceptible.

Our results were in agreement with a study reported by Singh et al.^17^ stated that *S.haemolyticus* (47.5 %) was the most common, *S.epidermidis* (33.9%) was the second most common, and 57.6% of all CNS were found to be resistant to methicillin. Bora et al.^1^ detected that *S.epidermidis*, *S.haemolyticus*, and *S.lugdunensis* are the most common bacteria in 120 CNS isolates, respectively. They observed an increase in the antibiotic resistance in penicillin and trimethoprim-sulfamethoxazole,and 100% sensitivity for linezolid. In our study, all 23 CNS isolates were found to be linezolid sensitive and penicillin-resistant, and 20 (87.0 %) were resistant or intermediately susceptible to trimethoprim-sulfamethoxazole. Czekaj et al.^18^ stated that *S. haemolyticus* isolates are more resistance to antimicrobials among CNS strains. In a study conducted in France in 2016^19^, the recovery rate of 88 patients with periprosthetic joint infection was found 89% in *S.lugdunensis*, 83% in *S.aureus*, and 97% in *S.epidermidis*. However, the resistance of *S. epidermidis* to methicillin, and clindamycin antibiotics has been reported to be much higher from two other bacteria and linezolid was sensitive in all of them. Our five *S.epidermidis* isolates were sensitive to linezolid but four were resistant to methicillin. In Korea, Shin et al.^20^ detected three *S.lugdunensis* among 358 CNS isolates from various clinical specimens. We observed that *S.haemolyticus* and *S.chromogenes* were more dominant in cancer and immunosuppressed patients. *S.haemolyticus* was found in the blood culture of four out of six isolated cancer patients (3 CML, one ALL, one recurrent ALL, and one Hodgkin lymphoma) and *S.chromogenes* was found in two of 11 cancer patients (seven with malignancy and four lung cancer). *S. epidermidis* was isolated from the blood of one out of five AML patients (Table-II).

### Limitations of the study

Low number of CNS srains that we obtained from 251 blood culture bottles was the main limitation of our study.

## CONCLUSION

We concluded that ODC and PYR tests are reliable tests and could be used successfully to identify *S.lugdunensis* and report correct AST results, and they are also capable of differentiating *S.epidermidis*, *S.haemolyticus*, and *S.chromogenes* from other CNS species. Linezolid was the most effective antibiotic.
